# Identification of a Cryptic Bacterial Promoter in Mouse (*mdr1a*) P-Glycoprotein cDNA

**DOI:** 10.1371/journal.pone.0136396

**Published:** 2015-08-26

**Authors:** Kristen M. Pluchino, Dominic Esposito, Janna K. Moen, Matthew D. Hall, James P. Madigan, Suneet Shukla, Lauren V. Procter, Vanessa E. Wall, Thomas D. Schneider, Ian Pringle, Suresh V. Ambudkar, Deborah R. Gill, Steven C. Hyde, Michael M. Gottesman

**Affiliations:** 1 Laboratory of Cell Biology, Center for Cancer Research, National Cancer Institute, National Institutes of Health, Bethesda, MD, United States of America; 2 Protein Expression Laboratory, Frederick National Laboratory for Cancer Research, Leidos Biomedical Research, Inc., Frederick, MD, United States of America; 3 Gene Regulation and Chromosome Biology Laboratory, National Cancer Institute, Molecular Information Theory Group, Frederick, MD, United States of America; 4 Gene Medicine Research Group, NDCLS, Radcliffe Department of Medicine, University of Oxford, Oxford, United Kingdom; University of Manchester, UNITED KINGDOM

## Abstract

The efflux transporter P-glycoprotein (P-gp) is an important mediator of various pharmacokinetic parameters, being expressed at numerous physiological barriers and also in multidrug-resistant cancer cells. Molecular cloning of homologous cDNAs is an important tool for the characterization of functional differences in P-gp between species. However, plasmids containing mouse *mdr1a* cDNA display significant genetic instability during cloning in bacteria, indicating that *mdr1a* cDNA may be somehow toxic to bacteria, allowing only clones containing mutations that abrogate this toxicity to survive transformation. We demonstrate here the presence of a cryptic promoter in mouse *mdr1a* cDNA that causes mouse P-gp expression in bacteria. This expression may account for the observed toxicity of *mdr1a* DNA to bacteria. Sigma 70 binding site analysis and GFP reporter plasmids were used to identify sequences in the first 321 bps of *mdr1a* cDNA capable of initiating bacterial protein expression. An *mdr1a* M107L cDNA containing a single residue mutation at the proposed translational start site was shown to allow sub-cloning of *mdr1a* in *E*. *coli* while retaining transport properties similar to wild-type P-gp. This mutant *mdr1a* cDNA may prove useful for efficient cloning of *mdr1a* in *E*. *coli*.

## Introduction

Since its identification nearly 35 years ago, the ATP-Binding Cassette (ABC) transporter P-glycoprotein (P-gp, ABCB1, MDR1) has been extensively studied and characterized. P-gp plays a vital role in the transport of molecules across cell membranes, recognizing a diverse range of substrates and using the energy from ATP hydrolysis to efflux molecules from cells, irrespective of the prevailing concentration gradient [1]. Physiologically, P-gp functions in both excretory and protective capacities by regulating concentration gradients of xenobiotics across biological membranes, being expressed in the liver, kidneys, lungs, and gastrointestinal tract and acting to efflux exogenous compounds and their metabolites into the bile, urine, mucus, and feces, respectively [2]. P-gp is also expressed at various physiological barriers, in endothelial cells of capillaries of the blood-brain barrier (BBB), the blood-cerebrospinal fluid barrier (CSF), and the blood-testis and blood-placental barriers [3,4]. P-gp recognizes a diverse range of substrates, including various therapeutically relevant natural product compounds, steroids, antiepileptic drugs, antibiotics, HIV protease inhibitors, immunosuppressive drugs, cardiac glycosides, and analgesics [5]. Thus, a consequence of these numerous roles is that P-gp can affect pharmacokinetic parameters of drug absorption, distribution, metabolism, excretion and toxicity [6]. Additionally, numerous anti-cancer drugs used in the clinic have been identified as substrates of P-gp, and overexpression of P-gp has been shown to correlate with overall poor chemotherapy response and prognosis and often prevents the successful treatment of cancer [5,7].

Full-length cDNAs of P-gp from various species are important for the functional characterization of human P-gp homologs. For example, *in vivo* models can be helpful to determine the biodistribution and metabolism of drugs in a pre-clinical setting, allowing drugs with undesirable pharmacokinetic parameters to be identified during drug development. However, these models are limited, as P-gp homologs from various species have shown both subtle and profound functional differences [8–13]. Additionally, several crystal structures of P-gp homologs, including those of mouse and *C*. *elegans* P-gp, have been recently reported, and a comprehensive understanding of functional differences between mouse and human P-gp homologs is required to understand both the impact and limitations of structural data [14–18]. P-gp cDNA from the species of interest are required to study functional differences between P-gp homologs in expression systems.

While P-gp cDNA is of critical importance for various experimental systems, propagation of mouse P-gp (*mdr1a*) cDNA in bacteria has proven difficult. Researchers in this laboratory and others have frequently observed the appearance of mutated *mdr1a* cDNA upon transformation into bacteria. This suggests *mdr1a* cDNA may be somehow toxic to *E*. *coli*, allowing only sub-clones with randomly occurring mutations in *mdr1a* cDNA that mitigate this selective pressure to survive transformation. Exogenous genetic material, such as cDNAs from eukaryotic organisms, may harbor sequences that are able to act as bacterial promoters and self-initiate their expression upon introduction into bacteria. Sequences from non-prokaryotic genomes that are able to induce transcription in a bacterial host are termed ‘cryptic promoters’ [19,20]. When present, cryptic promoters self-induce protein expression. Therefore, unintentional expression of plasmid cDNA may occur even in the absence of prokaryotic promoters in the plasmid vector. Deleterious effects on host bacteria as a result of protein expression resulting from a cryptic bacterial promoter have been previously reported with both mammalian and viral cDNAs [19,20]. Additionally, expression of eukaryotic membrane proteins (such as P-gp) in bacteria often leads to toxicity or cell death, as membrane-spanning domains can compromise the integrity of the cell membrane [21–23].

We hypothesized that the presence of a cryptic bacterial promoter in *mdr1a* cDNA, which results in the unintentional expression of P-gp, may account for observed difficulties in *mdr1a* propagation in bacteria. This study characterizes the genetic instability of *mdr1a* through transformation of *mdr1a* cDNA into *E*. *coli* and subsequent plasmid screening and sequencing to identify acquired mutations. Sigma 70 binding site analysis was used to identify the sequences of *mdr1a* cDNA capable of *mdr1a* transcription and translation in bacteria. Based on this analysis, GFP reporter constructs composed of N-terminal sequences of *mdr1a* fused to GFP were used to characterize the presence of a cryptic promoter. We discovered that sequences in the first 321 bps of *mdr1a* were able to express GFP. Lastly, an *mdr1a* cDNA M107L mutant that showed increased genetic stability was generated and functionally characterized.

## Materials and Methods

### Materials

All chemicals were sourced from Sigma Aldrich, St. Lois, MO unless otherwise stated.

### Bacterial and mammalian cell culture

Chemically competent One Shot Top10 *E*. *coli* cells were used for all sub-cloning and were cultured and transformed according to the manufacturer’s protocol (Life Technologies, Carlsbad, CA). Transformed *E*. *coli* cells were cultured with 100 μg/mL ampicillin, 50 μg/mL kanamycin, or 100 μg/mL zeocin depending on the antibiotic resistance gene contained in the plasmid backbone. The mammalian human embryonic kidney (HEK) 293 cell line was grown at 37°C in 5% CO_2_ and cultured in DMEM, supplemented with 10% fetal bovine serum, 5 mM L-glutamine, 50 units/mL penicillin, and 50 μg/mL streptomycin. Additionally, the human and mouse P-gp-expressing cell lines KB-8-5-11 and C3M were cultured in 100 ng/mL and 1 μg/mL colchicine, respectively, to maintain P-gp expression [24,25].

### Topo-cloning of PCR products

Cloning of agarose gel purified blunt-end PCR products was carried out using the Zero Blunt TOPO PCR Cloning Kit (Life Technologies, Carlsbad, CA, USA) into the supplied pCR-Blunt TOPO cloning plasmid according to the manufacturer’s protocols.

### Plasmids for GFP analysis

Plasmids were constructed using Multisite Gateway recombinational cloning (Life Technologies, Carlsbad, CA) using the manufacturer’s instructions. In each case, two separate Entry clones were recombined into the final Destination vector, pDest-302. This vector is a Gateway modified version of pUC19 and contains no prokaryotic promoter regions upstream of the cloning site. GFP plasmids contained the complete sequence of enhanced GFP (from pIRES2-eGFP) preceded by either a strong *E*. *coli* ribosome binding site and ATG start codon derived from pET43a (p300-GFP, p-GFP, λpR-GFP) or an in-frame tobacco etch virus protease cleavage site lacking an ATG start codon (p321-GFP, p321-M107L-GFP). The upstream *mdr1a*-containing Entry clones had regions of mouse *mdr1a* amplified from cDNA by PCR, and were fully sequence verified to ensure no mutations were introduced during amplification. The M107L mutation was introduced into one of these clones during amplification using a modified primer. The p-GFP stuffer region consisted of a portion of the *E*. *coli* chloramphenicol acetyl transferase gene which lacked prokaryotic promoter activity, had no ATG codons, and had stop codons present in all reading frames to ensure no transcriptional or translational activity. The λpR-GFP region contained a 203 bp region of the bacteriophage lambda early DNA which encodes the highly active phage pR promoter (Genbank Refseq NC_001416).

### Plasmids for HEK-293 transfection

Full length human P-gp was amplified by PCR from cDNA and used to construct a Gateway Entry clone that was fully sequence verified. Mouse M107L P-gp was generated by overlap extension PCR from cDNA constructs and used to construct a Gateway Entry clone, which was also fully sequence verified. Both Entry clones were sub-cloned into pDest-780, a Gateway modified version of pcDNA3.2 (Life Technologies, Carlsbad, CA), which allows expression of the gene of interest with the strong CMV promoter.

### Lipofectamine transfection

Lipofectamine transfections were performed using Lipofectamine 2000 Reagent according to the manufacturer’s protocol (Life Technologies, Carlsbad, CA). Briefly, 5×10^5^ HEK-293 cells were transfected with 2 μg of plasmid DNA. HEK-293 cells were incubated for 24 hours, after which media was replaced with media containing 100 nM paclitaxel and incubated for an additional 48 hours before harvesting.

### Purification of plasmid DNA

For small-scale plasmid purification, 2–5 mL of culture was purified using the Wizard Plus SV Miniprep DNA Purification system (Promega, Madison, WI) according to the manufacturer’s protocol. For large-scale plasmid purification, 200–500 mL of culture was purified using the HiSpeed Plasmid Maxi Kit (Qiagen, Limburg, Netherlands) according to the manufacturer’s protocol.

### Sequencing and sequence analysis of DNA

Sequencing was carried out using Big Dye Terminator sequencing kits (Life Technologies, Carlsbad, CA). Analysis of sequencing chromatograms was carried out using MacVector alignment software (MacVector, Cary, NC). Sequencing chromatograms of wild-type *mdr1a* and *MDR1* were aligned to reference sequences from Genbank identifications NM_011076.2 (*mdr1a*) and NM_000927.4 (*MDR1*).

### Fluorescence spectroscopy

Fluorescence spectroscopy was used to determine levels of GFP fluorescence in bacteria transformed with GFP plasmids. One Shot Top10 Chemically Competent *E*. *coli* cells were transformed with GFP plasmids, and transformants were plated on LB agar containing the appropriate antibiotic selective agent. Colonies were selected for growth in 5 mL of LB media for 12 hours. Cultures were centrifuged and LB media was removed. Cell pellets were resuspended in PBS and transferred to a 96-well, black walled plate for fluorescence analysis. Fluorescence intensity was measured by the FLUOstar Omega fluorometer (BMG Labtech, Ortenberg, Germany).

### Confocal microscopy

Transformed *E*. *coli* to be analyzed for GFP fluorescence by confocal microscopy were transferred to chambered coverslips. Cells were imaged with a Zeiss LSM 710 NLO confocal microscope and images were processed by Zen 2012 software (Zeiss, Jena, Germany).

### Western blotting

Samples were prepared by combining the desired amount of protein with 1x SDS sample buffer and loaded into each well of a Bis-Tris SDS-PAGE gel and electrophoresed at 150 V in 1x MOPS running buffer (Life Technologies, Carlsbad, CA). Protein was transferred to a PVDF membrane using the iBlot Dry Blotting System (Life Technologies, Carlsbad, CA). Membranes were blocked in 10% non-fat milk for at least 1 hour. After blocking, membranes were incubated with either the C219 (Fujirebio Diagnostics, Malvern, PA) or GAPDH primary antibody for 1 hour. Membranes were washed and incubated with the anti-mouse IgG HRP-linked secondary antibody for an additional hour before addition of chemiluminescent horseradish peroxidase (HRP) antibody detection reagent and exposure to chemiluminescence film. All antibodies in this study were used at a 1:10,000 dilution.

### ATPase assay

ATPase assays were carried out using membranes prepared from baculovirus-infected High-five insect cells as previously described [8,26]. Membrane vesicles (10 μg of protein) were incubated with varying concentrations of the drug to be tested in the presence and absence of 0.3 mM sodium orthovanadate in ATPase 2x assay buffer (100 mM MES-Tris buffer (pH 6.8), 100 mM KCl, 10 mM sodium azide, 2 mM EGTA, 2 mM ouabain, 20 mM MgCl2, and 4 mM DTT) for 5 min. The reaction was started by the addition of 5 mM ATP. Each assay condition was allowed to incubate at 37°C for 20 minutes before the addition of 100 μL of 5% SDS. To determine the release of inorganic phosphate, 500 μL of deionized water, 400 μL of P_i_ reagent (composed of 1% ammonium molybdate in 2.5 M sulfuric acid and 0.014% potassium antimonyl tartrate trihydrate) and 200 μL of 1% ascorbic acid was added to each tube. Color was allowed to develop for 10 minutes before the absorbance was measured spectrophotometrically at 880 nm. Absorbance values were used to calculate the rate of ATPase specific activity (nmoles P_i_/min/mg protein).

### Flow cytometry P-gp transport function assay

Cells were trypsinized, counted, and 2 × 10^5^ cells per assay conditions were transferred into polystyrene flow cytometry tubes. Cells were incubated in IMDM containing 0.5 μM Rhodamine 123 (Rh123) with or without 100 nM of the P-gp inhibitor tariquidar at 37°C for 45 minutes. After incubation, cells were centrifuged, supernatant was removed, and cells were resuspended in 200 μL of cold PBS and placed on ice to stop transporter activity. A total of 10,000 individual cell-counting events were recorded using a FACSCanto II flow cytometer. A 488 nM blue laser was used to excite all fluorophores and emission was measured at 530 nm. FACS data were analyzed using FlowJo Single Cell Analysis software.

### Sigma 70 binding site analysis

Promoters and ribosome binding sites were identified using sequence walkers as previously described [27,28].

### Statistical analysis

Curve-fitting and all statistical analysis was performed using GraphPad Prism 6.0. Multiple groups were analyzed using one-way analysis of variance (ANOVA) where significance was defined as p < 0.05.

## Results

### Characterization of *mdr1a* cDNA genetic instability in bacteria

**Fig 1:**
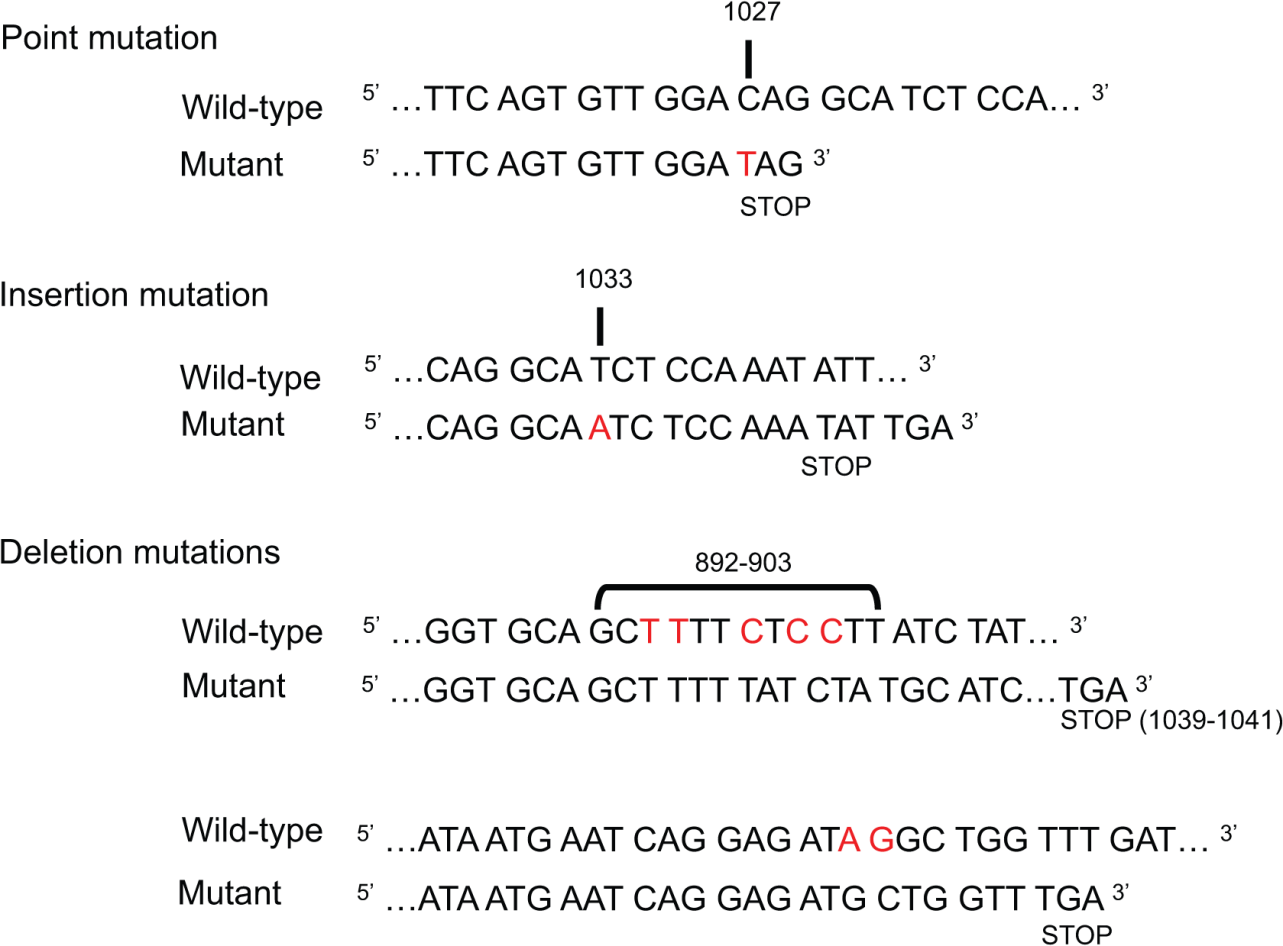
Mutations and their position in *mdr1a* cDNA after transformation into *E*. *coli*. The pCR-Blunt-II-TOPO vector containing *mdr1a* cDNA was used to transform *E*.*coli* and 20 colonies were selected for small-scale bacterial growth, plasmid purification, and analysis of plasmid DNA by agarose gel electrophoresis. Sixteen of the colonies selected for analysis contained large insertion or deletion mutations (not shown), and the remaining four colonies containing potentially correct *mdr1a* cDNA were sequenced. Each of the four sequenced plasmids contained mutated *mdr1a* cDNA, indicating a 100% mutational rate. Mutations observed were classified as point, insertion, or deletion mutations. Wild-type *mdr1a* cDNA (top) is compared to sequenced, “mutant” *mdr1a* cDNA (bottom). Insertion and point mutations are indicated in red in the mutant cDNA. Deleted base pairs are highlighted in red in wild-type cDNA, and the resulting mutated cDNA sequence is shown below. A) A point mutation (C→T) at location 1027 bp resulting in an introduced stop codon into *mdr1a* cDNA. B) A single adenine base pair insertion at location 1033 bp. C) Two examples of deletion mutations. All mutations resulted either directly (point mutations) or indirectly (insertion or deletion mutations) in the introduction of a stop codon into *mdr1a* cDNA. Locations of the introduced stop codons are indicated.

Plasmids containing *mdr1a* cDNA were constructed by PCR amplification of mouse *mdr1a* cDNA and ligation into the pCR-Blunt-II-TOPO vector using Zero Blunt TOPO PCR Cloning. Twenty clones were selected for plasmid purification and analysis by gel electrophoresis. Typically, 5% of all Zero Blunt TOPO PCR Cloning reactions result in the self-ligation of the vector without the PCR product of interest. However, when purified plasmids were analyzed for size by gel electrophoresis, only four of 16 plasmids displayed the ligation of *mdr1a* cDNA into the pCR-Blunt-II-TOPO vector. Each of the four potential *mdr1a*-containing plasmids were sequenced (Fig 1). Three plasmids contained *mdr1a* cDNA with frameshift mutations (insertion or deletion mutations that would generate an out-of-frame protein if expressed). One contained a single point mutation that introduced a stop codon into *mdr1a* cDNA. These data indicate the presence of wild-type *mdr1a* cDNA in bacteria is intolerable, allowing only bacteria containing plasmids with mutated *mdr1a* cDNA to survive transformation.

To investigate whether mutations to *mdr1a* cDNA were specific to the Zero Blunt TOPO PCR Cloning system, *mdr1a* cDNA was ligated into an alternative vector for transformation into *E*. *coli*. The pPICZ vector was chosen, as *mdr1a* in this vector has been used to express mouse P-gp in the yeast *Pichia pastoris* for subsequent crystallographic studies, and plasmid amplification was carried out in *E*.*coli* [14,29]. Interestingly, *mdr1a* cDNA in the pPICZ plasmid was recovered without mutation on occasion. However, *mdr1a* in the pPICZ vector was also prone to various mutations. For example, two plasmids selected for further analysis acquired insertions of approximately 1.3 and 0.7 kb. Sequence analysis was undertaken and the inserted sequences showed sequence homology to the bacterial insertion elements IS10 and IS1. Thus, the high mutation rate observed with *mdr1a* cDNA in different plasmid backbones indicates a toxic element intrinsic to *mdr1a* cDNA.

### *In silico* identification of a cryptic promoter in *mdr1a* cDNA

To investigate the possible presence of a cryptic promoter in *mdr1a* cDNA, *in silico* sigma 70 binding site analysis using information theory was used to search for potential bacterial promoters [27]. Additionally, information theory can identify sequences capable of initiating translation by identifying sequences similar to the bacterial ribosome binding site (RBS), which consists of both the Shine-Dalgarno sequence and the initiation codon [28,30,31]. By the identification of predicted transcriptional and translational initiation sites, potential open reading frames (ORF) can be detected.

**Fig 2:**
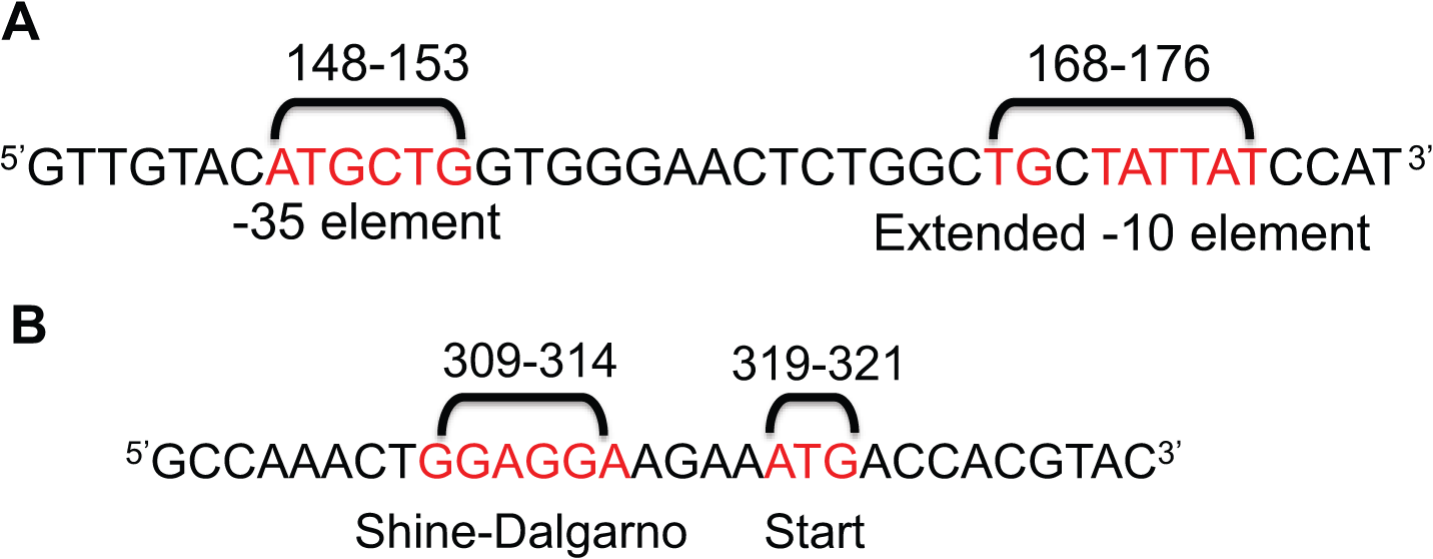
Identification of putative transcriptional and translational start sites in *mdr1a* cDNA. An *in silico* sigma 70 binding site analysis was used to screen for a putative cryptic bacterial promoter in *mdr1a* cDNA. (A) Sequence of *mdr1a* cDNA shown with predicted -35 and extended -10 elements (red). The location in the cDNA for each element is noted above the sequence. (B) Predicted Shine-Dalgarno and ATG of a methionine that is in-frame with regards to the *mdr1a* ORF.

The sigma 70 binding site analysis identified a sequence in *mdr1a* cDNA capable of acting as a bacterial promoter (Fig 2A and S1 Fig). This sequence consists of a classic extended -10 element (TGnTATAAT) located between 168–176 bps of *mdr1a* cDNA. The analysis did not predict the presence of a -35 element capable of promoter activity. However, previous studies have shown that the presence of an extended -10 element is capable of high levels of protein expression, even in the absence of additional promoter elements (-35 element/UP element) [32]. Thus, it is likely that the extended -10 element is responsible for transcription of *mdr1a* cDNA.

While this promoter is theoretically capable of initiating transcription, to support the hypothesis that *mdr1a* toxicity is due to the expression of P-gp, a RBS and translation start site must also be present in the cDNA. The analysis identified a RBS ideally spaced upstream of a methionine start (ATG) codon (Fig 2B). Additionally, the methionine is in-frame with regard to the ORF of *mdr1a* cDNA. In other words, if translation were initiated at this location, P-gp would be expressed in *E*. *coli* initiating at amino acid 107 and terminating at the native stop codon after amino acid 1276. Additionally, *MDR1* human P-gp cDNA (*MDR1* is 83% identical to *mdr1a* cDNA) was also analyzed by sigma 70 binding site analysis for the presence of a cryptic promoter, as this laboratory has not observed genetic instability of *MDR1* cDNA. It did not predict that the corresponding *MDR1* sequence would act as a bacterial promoter due to differences in the analogous sequence of the predicted -10 element, a fact that may explain why genetic instability is observed with mouse, but not human, P-gp cDNA (data not shown).

### Sequences of *mdr1a* cDNA are able to drive GFP expression

**Fig 3:**
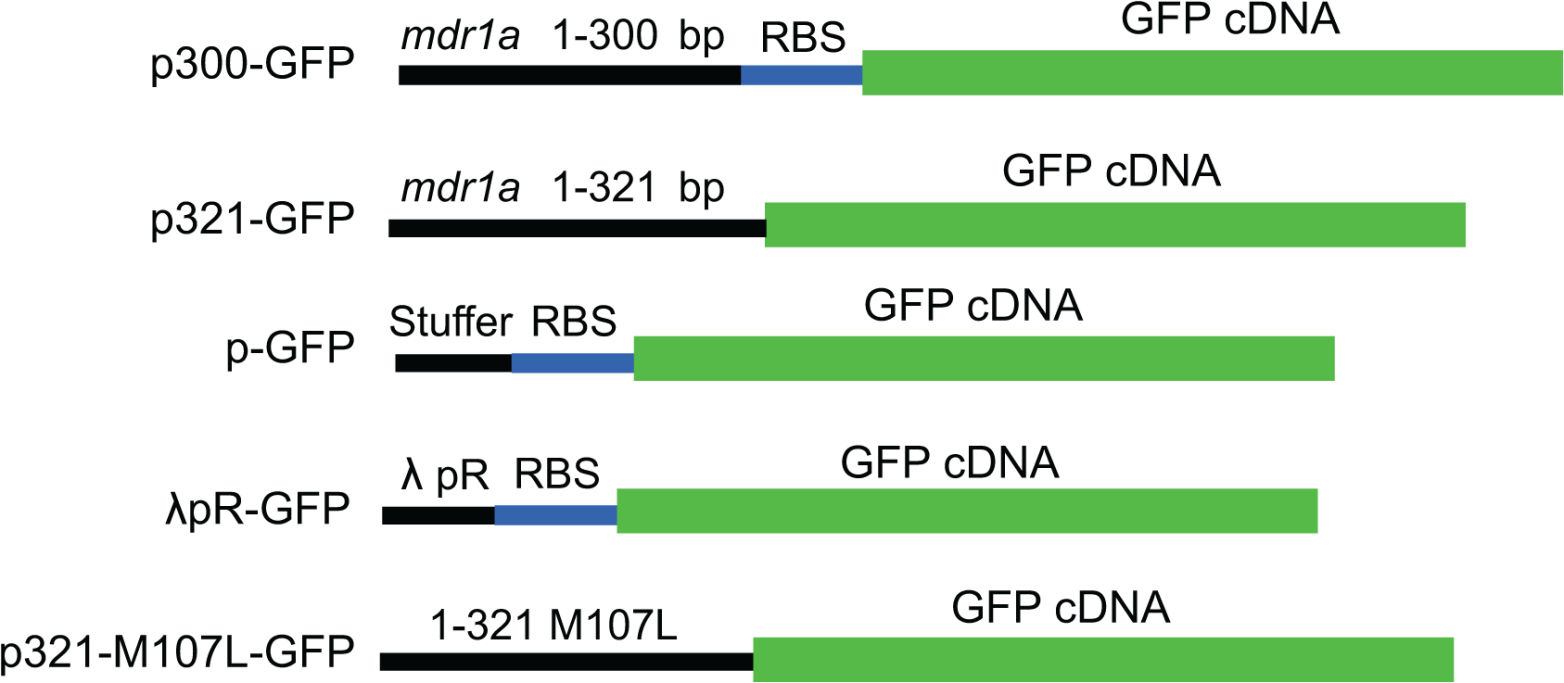
Schematic of GFP fusion constructs. GFP reporter constructs were generated to assess the ability of sequences of *mdr1a* cDNA to drive protein expression. Black lines represent DNA sequences fused upstream of GFP. GFP cDNA is represented in green and the presence of introduced RBSs are shown in blue where applicable. The p300-GFP plasmid contains the first 300 bps of *mdr1a* fused to GFP immediately preceded by a strong RBS. The p321-GFP plasmid contains the first 321 bps of *mdr1a* fused to GFP without the presence of an introduced RBS. pGFP and λpR-GFP were used as positive and negative control for GFP expression, respectively. p321-GFP and p-321-M107L-GFP are equivalent with the exception of the methionine to leucine mutation at position 107 in the amino acid sequence.

To investigate the cryptic promoter predicted by the sigma 70 binding site analysis, a series of plasmids were generated containing truncations of *mdr1a* cDNA fused to GFP (Fig 3). Plasmids were designed to investigate several aspects of the ability of *mdr1a* cDNA to initiate transcription and translation in bacteria. As the *in silico* analysis predicted a bacterial promoter between 168 and 176 bp, plasmid p300-GFP was generated to determine the ability of sequences in the first 300 bps of *mdr1a* cDNA to promote GFP expression. Note that this construct contains an introduced RBS and ATG initiation codon to initiate GFP translation, and is therefore only reporting on the ability of the first 300 bps of *mdr1a* cDNA to function as a bacterial promoter. In addition to a cryptic promoter that would initiate transcription, translation must also occur for the expression of mouse P-gp. The GFP fusion p321-GFP was generated to investigate the ability of *mdr1a* to express GFP. Plasmid p321-GFP is composed of *mdr1a* cDNA 1–321 bp, which includes the predicted cryptic promoter (*mdr1a* bases 168–176), RBS (*mdr1a* bases 309–314), and the mouse P-gp methionine at amino acid 107 (*mdr1a* bases 319–321) fused in-frame with GFP lacking a start codon. GFP expression observed from this construct after transformation into bacteria would suggest that both the transcriptional and translational sequences identified by the *in silico* analysis are functional.

Additionally, p321-M107L-GFP was constructed to encode a leucine rather than a methionine at position 107 in the amino acid sequence. This was generated in an attempt to abrogate GFP expression by removing the translational start site, and investigate if construction of the mutated form of *mdr1a* harboring an M107L mutation would be desirable. Plasmid p-GFP, containing a stuffer region in lieu of a promoter, and λpR-GFP, containing the strong λpR promoter, were generated as controls.

**Fig 4:**
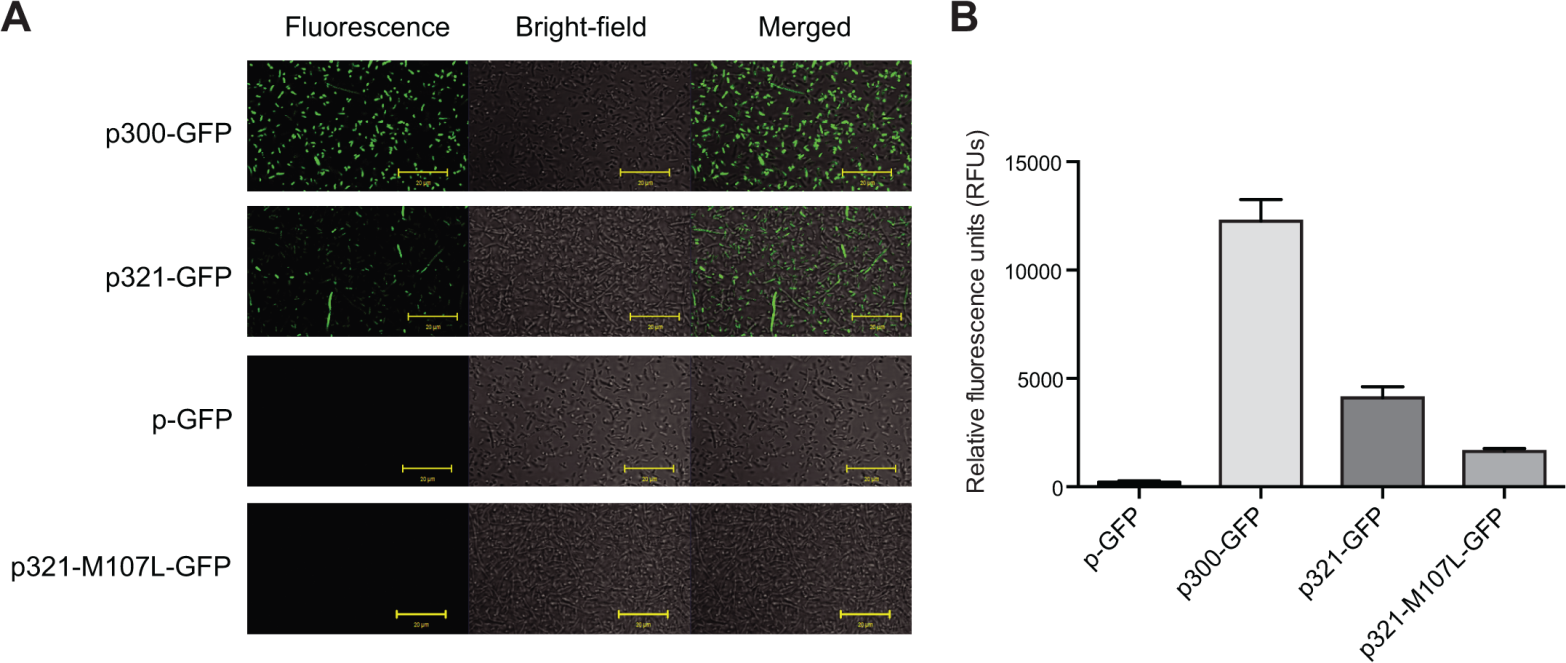
Fluorescence of *E*. *coli* transformed with GFP fusion plasmids. (A) Confocal images of *E*. *coli* transformed with GFP fusion plasmids. From left to right, columns represent fluorescence, bright field, and a merged image. Yellow bars indicate 20 μm. (B) Fluorescence of *E*. *coli* transformed with GFP fusion plasmids. Fluorescence spectroscopy was used to determine levels of GFP fluorescence in bacteria transformed with GFP plasmids. Confocal images and fluorescence spectroscopy values for bacteria transformed with λpR-GFP plasmids were omitted due to the signal saturation resulting from high GFP fluorescence. Data represent the mean and standard deviation of five individual colonies from one transformation event. Multiple groups were analyzed using one-way ANOVA where significance was defined as p < 0.05 in GraphPad Prism.

The p300-GFP, p321-GFP, p321-M107L-GFP, λpR-GFP, and p-GFP plasmids were used to transform *E*. *coli*. Confocal microscopy was used to qualitatively determine levels of GFP expression in bacteria transformed with the p300-GFP, p321-GFP, p321-M107L-GFP, λpR-GFP, and p-GFP plasmids (Fig 4A). Bacteria transformed with the p300-GFP and p321-GFP plasmids exhibited GFP expression. Confocal images of transformed bacteria were in qualitative agreement with fluorescence levels observed by fluorescence spectroscopy used to quantify the amount of GFP expression in transformed bacteria (Fig 4B). Bacteria transformed with the p300-GFP plasmid displayed significantly higher GFP expression as compared to p-GFP as seen by the increase in fluorescence intensity from 218 ± 61 RFU (p-GFP) to 12,255 ± 1,005 RFU (p300-GFP) (p ≤ 0.0001), indicating that the *mdr1a* cDNA sequence from 1–300 bp contains promoter elements able to initiate GFP transcription. The p321-GFP plasmid was used to determine if sequences 1–321 bp of *mdr1a* were able to express GFP by both the production of a transcript and subsequent translation into a protein product. Fluorescence intensity of the bacteria transformed with p321-GFP (4,110 ± 515 RFU) was also significantly higher than p-GFP (p ≤ 0.0001). However, p321-GFP was also significantly lower than observed with p300-GFP (p ≤ 0.0001). This is most likely due to the fact that the p300-GFP plasmid contains an introduced strong RBS with ideal positioning relative to the transcriptional start site. In contrast, the p321-GFP is predicted by the sigma 70 binding site analysis to contain a weaker RBS with less ideal spacing between the RBS and the transcriptional start site. Confocal images and fluorescence spectroscopy values for bacteria transformed with λpR-GFP plasmids were omitted due to the signal saturation resulting from high GFP fluorescence.

GFP fusion plasmid p321-M107L-GFP containing the M107L mutation had significantly lower GFP expression (1,636 ± 129 RFU) than bacteria transformed with the p300-GFP (12,255 ± 1,005 RFU) and p321-GFP (4,110 ± 515 RFU) plasmids (p ≤ 0.0001), indicating that introduction of the M107L mutation to full-length *mdr1a* may reduce toxicity and generate a more stable *mdr1a* cDNA. However, the M107L mutation did not completely abrogate GFP expression, as GFP levels in bacteria transformed with p321-M107L-GFP (1,636 ± 129 RFU) contained higher GFP expression than bacteria transformed with the negative control p-GFP (218 ± 61 RFU) plasmid DNA (p ≤ 0.01). Various explanations could account for GFP expression by the p321-M107L-GFP plasmid, such as the presence of another codon that may be able to initiate translation. For example, while ATG is the most common start codon, used to initiate translation in approximately 83% of genes in *E*. *coli*, GTG and TTG initiate translation in 14% and 3% of genes, respectively [33]. Additionally, translation of at least one gene in *E*. *coli* uses CTG (the codon used to generate the leucine mutation) as a start codon for translation [34].

### *mdr1a* M107L cDNA shows increased genetic stability during cloning

While *mdr1a*-GFP fusion plasmids containing the M107L mutation had significantly less GFP expression, the critical question was whether the M107L mutation would reduce *mdr1a* cDNA-associated toxicity. Full-length *mdr1a* cDNA was mutated to introduce the M107L mutation to generate *mdr1a*_M107L cDNA. The *mdr1a*_M107L cDNA was ligated into a pDest-008 plasmid to generate the pDEST_*mdr1a*_M107L plasmid, which was used to transform *E*. *coli*. Three colonies were selected for small-scale culturing and subsequent plasmid purification and sequencing. All three pDEST_*mdr1a*_M107L purified plasmids contained non-mutated cDNA (apart from the introduced M107L mutation). Elimination of the translational start site associated with the cryptic bacterial promoter appeared to reduce the toxicity of *mdr1a* cDNA to bacteria.

### Functional characterization of M107L P-gp

**Fig 5:**
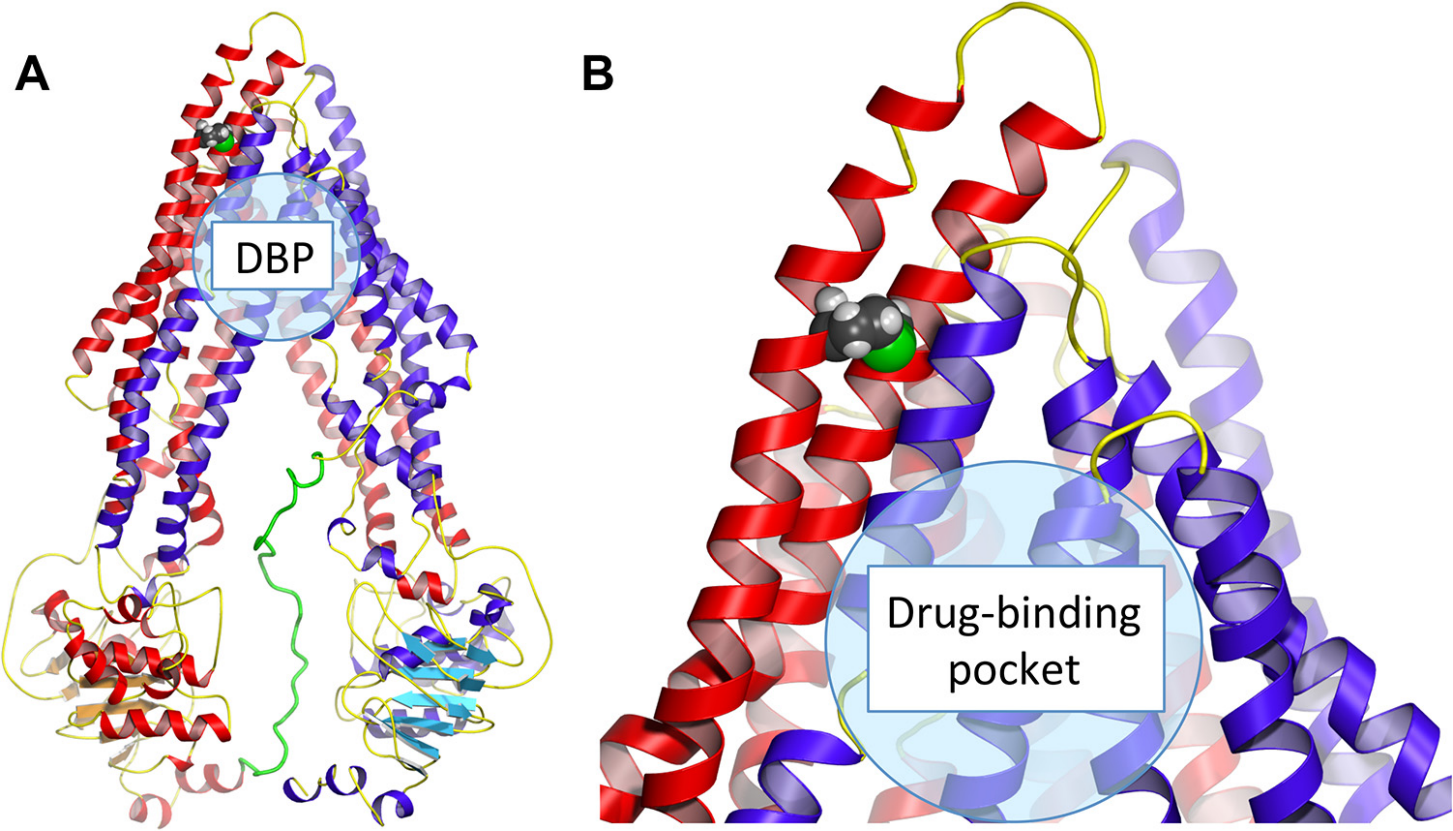
Mouse P-gp model in apo (open) conformation with methionine residue at position 107 highlighted. (A) Mouse P-gp is shown with the methionine to leucine mutation highlighted in TM1-ECL1 by the presence of a space filled leucine residue. The region of the drug-binding pocket (DBP) is indicated. (B) Enlarged view of M107L mutation. Figures were generated using PyMOL from PDB deposit 4M1M [16].

To evaluate if a mutation at residue 107 in *mdr1a* would be likely to affect protein function, the residue was located in the mouse P-gp crystal structure (Fig 5). Residue 107 is located at the boundary of extracellular loop 1 (ECL1) and transmembrane helix 1 (TM1). In this model, residue 107 does not face the drug-binding pocket, and would not be expected to influence function. Leucine was chosen as a replacement residue for methionine, as both are residues with hydrophobic side chains that are relatively similar in size, and the codon for leucine (CTG) is not expected to efficiently initiate translation.

To show that M107L P-gp was functionally similar to wild-type P-gp, complete functional characterization was initiated. Characterization of M107L P-gp was carried out in comparison to human P-gp. A more accurate assessment of M107L function would be to compare the functional activity of M107L P-gp to wild-type mouse P-gp, as species differences may occur naturally between mouse and human P-gp, and these variables may confound functional differences resulting from the M107L mutation. However, due to mutations in wild-type *mdr1a* cDNA acquired during cloning, the plasmids required to carry out functional assays could not be generated. Therefore, all functional assays carried out used human P-gp as a control for M107L P-gp expression and function.

**Fig 6:**
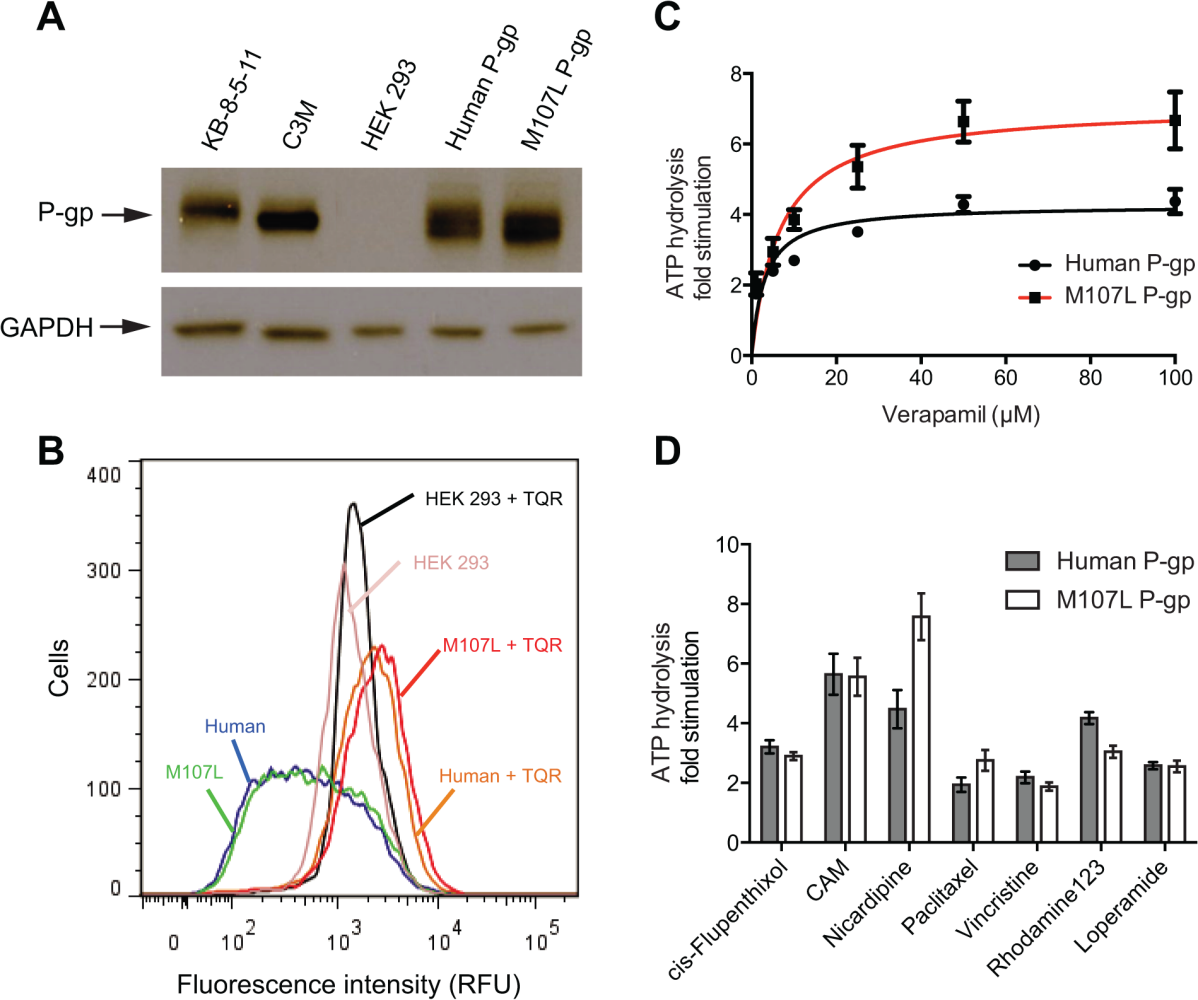
Functional characterization of M107L P-gp. (A) HEK-293 cells were transiently transfected with plasmids containing human and M107L P-gp for subsequent western blotting with the C219 primary antibody. Additionally, the drug-selected cell lines C3M and KB-8-5-11 over-expressing wild-type mouse and human P-gp, respectively, were included. GAPDH was used as a loading control. (B) Rh123 efflux assay of HEK-293 cells transfected with human and M107L P-gp. Histograms shown are representative of three independent studies. (C) ATPase assays were carried out using crude membranes generated from High-five insect cells infected with recombinant baculovirus expressing human and M107L P-gp. ATPase activity of human WT and M107L P-gp in the presence of 0, 1, 5, 10, 25, 50 and 100 μM of verapamil. (D) Seven known stimulators of ATPase activity were selected to determine their effect on the activity of M107L P-gp compared to human WT P-gp. The following concentrations were used of each compound: 20 μM cis-flupentixol, 10 μM CAM, 20 μM nicardipine, 20 μM paclitaxel, 20 μM vincristine, 500 μM Rhodamine 123, and 20 μM loperamide. All ATPase data represent average and standard deviation of technical duplicates from three individual assays.

Vectors containing human P-gp cDNA and mouse P-gp cDNA with the M107L mutation (M107L P-gp) were used to transiently transfect HEK-293 cells for western blotting and flow cytometry studies. Western blotting was performed to determine if M107L P-gp was expressed at a level comparable to wild-type human P-gp. The C219 primary antibody was used for western blotting, as it recognizes an epitope conserved in all mammalian P-gps, including mouse and human P-gp [35]. HEK-293 cells transfected with human and M107L P-gp both expressed P-gp to equivalent levels (Fig 6A), whereas P-gp was not detected in untransfected HEK-293 cells.

Flow cytometry efflux assays were carried out to determine functionality of M107L P-gp compared to human P-gp. Rhodamine 123 (Rh123) is a fluorescent compound that is a substrate of mouse and human P-gp. HEK-293 cells transfected with human and M107L P-gp should efflux equivalent amounts of Rh123. Non-transfected HEK-293 cells should accumulate high intracellular levels of Rh123, as these cells lack P-gp-mediated efflux. The extent of P-gp-mediated efflux can be observed by the co-incubation of transfected cells with both Rh123 and the P-gp inhibitor tariquidar (TQR). HEK-293 cells transfected with human and M107L mouse P-gp effluxed Rh123 to a similar extent, as indicated by the low level of cellular fluorescence (Fig 6B). Non-transfected HEK-293 cells, which do not express P-gp, accumulated high levels of Rh123. Additionally, when cells transfected with human or M107L P-gp were co-incubated with Rh123 and TQR, P-gp-mediated efflux was abrogated, allowing Rh123 to accumulate in cells, as observed by the increased intracellular fluorescence to non-transfected levels. Thus, M107L P-gp was shown to be expressed and functional at a level similar to that of wild-type human P-gp.

ATPase activity is required for the transport of substrates by P-gp. Therefore, ATPase assays were carried out to determine biochemical differences in the ATP binding and hydrolysis between human and M107L P-gp. Various concentrations of verapamil were tested to measure ATPase activity of human and M107L P-gp (Fig 6C). Interestingly, M107L P-gp exhibited higher maximal fold stimulation than human P-gp. As the M107L mutation is not located in the drug-binding pocket and therefore not predicted to influence drug transport (Fig 5), this result may reflect a species difference between mouse and human P-gp. To determine if differences in ATPase activity were observed with additional compounds, a panel of seven compounds known to stimulate P-gp ATPase activity was screened (Fig 6D). The compounds cis-flupentixol, calcein AM (CAM), nicardipine, paclitaxel, vincristine, Rh123, and loperamide were used to probe for activity of M107L P-gp compared to human P-gp. Human and M107L P-gp showed similar levels of ATPase stimulation, indicating that M107L P-gp is functional and its ATPase activity can be stimulated to levels similar to those of human P-gp with the exception of nicardipine and verapamil stimulation. Overall, while M107L P-gp displayed higher levels of ATPase activity with certain substrates, M107L P-gp functionality was deemed similar to that of wild-type P-gp.

## Discussion

The heterologous expression of some eukaryotic membrane proteins in bacteria can exert toxic or lethal effects due to perturbation of the bacterial cell membrane by the insertion of transmembrane-spanning domains [21–23]. However, to our knowledge, plasmids containing *mdr1a* cDNA did not contain a bacterial promoter upstream of *mdr1a*, and thus protein expression or subsequent toxicity was not expected to occur. This study identifies and characterizes a cryptic bacterial promoter intrinsic to mouse *mdr1a* cDNA capable of protein expression. The genetic instability of plasmids containing *mdr1a* cDNA had been previously observed by several laboratories [36,37], but no study has formally reported the detrimental effects of plasmids containing *mdr1a* cDNA upon transformation into *E*. *coli* or characterized the basis of such effects. The experiments described in this study have identified the presence of sequences intrinsic to *mdr1a* cDNA that are able to initiate transcription and translation and thus induce protein expression. Additionally, this study reports a *mdr1a* M107L mutant generated by a single point mutation that is more genetically stable during cloning and retains the functional characteristics of wild-type P-gp.

The toxicity of foreign DNA in bacteria and the mechanism by which this occurs has been studied and often found to be due to cryptic promoters. This is a form of the ‘pervasive transcription’ reported to occur in the bacterial genome. While bacteria do have machinery to suppress pervasive transcription (binding by histone-like nucleoid protein, H-NS), this largely occurs against genes that are A/T-rich (*mdr1a* cDNA is unremarkable at 54% AT-rich) [38]. The ABC transporter CFTR (ABCC7) provides a notable example of the cryptic promoter phenomenon [39]. A seminal paper by Gregory *et al*. characterized such a cryptic bacterial promoter in CFTR cDNA [19]. The authors observed that *E*. *coli* transformed with plasmids containing human CFTR cDNA often yielded plasmids with extensive rearrangements, indicating that transformed bacteria might be selecting against propagation of the full length CFTR cDNA. To investigate if any portion of the CFTR cDNA was being unintentionally expressed due to the presence of a cryptic bacterial promoter, plasmids containing segments of CFTR fused to chloramphenicol acetyltransferase (CAT), a gene that confers resistance to chloramphenicol, were generated. The authors reported a region of CFTR cDNA between 908 and 936 bp that was able to generate chloramphenicol-resistant *E*. *coli* clones due to the expression of CAT. To abrogate protein expression of CFTR cDNA in *E*. *coli*, a T936C silent mutation was introduced into the identified -10 promoter element, therefore generating a cDNA mutant capable of propagating in bacteria [40–43]. More recently, a cryptic bacterial promoter was characterized in the cDNA encoding the Dengue virus (DENV) 5’ untranslated region (UTR) [20]. Li *et al*. described the high mutation rate of plasmids containing DENV 5’UTR cDNA and identified sequences of cDNA highly homologous to bacterial -35 and -10 promoter elements that were able to express a toxic protein product.

Several early papers used cloning as a method for the propagation and amplification of plasmids containing *mdr1a* [12,44,45]. Unfortunately, published literature often did not report the exact cloning conditions (bacterial host strain used, bacterial growth conditions, etc.) and it was not possible to repeat the cloning strategy employed. It may be that various factors that allowed for the cloning of *mdr1a* in those early reports are no longer applicable. For example, plasmids used in earlier studies are now often considered to be ‘low-copy number vectors’, with plasmids present in bacterial cells in lower numbers than current commercially available plasmid vectors optimized for high plasmid yield; and lower plasmid copies per cell would be expected to decrease the deleterious effects of a cryptic promoter if present in *mdr1a*. Also, at their time of publication, sequence analysis was not standard practice, and it is possible that the constructs described may have contained unidentified compensatory mutations. Nonetheless, the limited published literature on this subject does not describe or characterize difficulty in cloning *mdr1a*.

A series of papers by Bibi *et al*. report methods for the expression of *mdr1b* (also termed mdr1), which is 83% homologous to *mdr1a*, using *E*. *coli* [36,46,47]. This suggests that, even if *mdr1a* was expressed in the presence of a cryptic promoter, the protein product would not be harmful to bacteria [48]. However, close inspection of these papers reveals multiple insights that would suggest the converse is true. For example, the authors screened a variety of bacterial host strains to identify a strain that was able to withstand *mdr1b* expression [36]. Additionally, the authors noted that high-copy number plasmids were not suitable for the expression of *mdr1b*, stating that high copy vectors increase the toxic effects of *mdr1b* in bacteria [36]. The authors also noted the presence of two distinct colony morphologies after bacterial transformation with plasmids containing *mdr1b*: large colonies that contained mutated *mdr1b* and small, slow-growing colonies containing wild-type *mdr1b*. Taken together, these data suggest that while certain bacterial strains and growth conditions may allow for the expression of *mdr1b*, its expression does exert a toxic effect when expressed in bacteria. Lastly, the authors attempted the expression of *mdr1a* in *E*. *coli* with various host strains, but several rearrangements of *mdr1a* cDNA were observed and expression could not be obtained (E. Bibi personal communication, Weizmann Institute of Science).

Lastly, a paper by Evans *et al*. describes attempts made to express human P-gp in *E*. *coli* for structural investigations where P-gp expression was deliberately driven by *lac* operon induction [37]. Expression of full-length human P-gp could not be attained. However, low-level expression of a truncated form of P-gp was detectable, and expression of the truncated protein was associated with a 500- to 1000-fold drop in colony forming ability [37]. Taken together with the current study, these data indicate that *E*. *coli* is not able to withstand the expression of either human or mouse P-gp; however, expression of human P-gp only occurs through plasmid-driven induction, while the expression of mouse P-gp occurs unintentionally through the activity of a cryptic promoter.

A thorough understanding of functional differences between mouse and human P-gp is a crucial step towards both the translation of *in vivo* animal studies to the clinic and a comprehensive understanding of the recently reported mouse P-gp crystal structures. To date, a severe impediment to the functional characterization of mouse P-gp has been the inability to reliably clone plasmids containing *mdr1a* cDNA. We hope that this study provides a warning to researchers working with *mdr1a* concerning its propensity to mutate during cloning. Our discovery of a cryptic promoter present in *mdr1a* is a possible explanation for the observed genetic instability of the plasmid. We believe that the M107L *mdr1a* mutant characterized here, which is capable of replication in *E*. *coli* with a reduced mutational rate, may prove useful for laboratories wishing to study *mdr1a* in the future.

## Supporting Information

S1 Fig Putative sigma 70 promoter and ribosome binding sites in mouse *mdr1a* cDNA. The coordinate system begins at the first base of the *mdr1a* coding region. For the first two sequences, flexible individual information models for *E*. *coli* sigma70 (yellow, red rectangular petals) and extended -10 sigma70 (green) and ribosome binding sites (purple and blue) were scanned over the sequence and displayed using sequence walkers. The third sequence shows the effect of changing base 319 from an A to a C in M107L. This lowers the initiation codon from 6.3 bits to -5.5 bits, effectively removing the ribosome binding site.
(PDF)
