# Supplementary material for: Identification of a Cryptic Bacterial Promoter in Mouse (mdr1a) P-Glycoprotein cDNA
Source: PLoS One. 2015 Aug 26;10(8):e0136396. doi: 10.1371/journal.pone.0136396 (PMC4550409; doi:10.1371/journal.pone.0136396)
Supplement: S1 Fig — The coordinate system begins at the first base of the mdr1a coding region. For the first two sequences, flexible individual information models for E. coli sigma70 (yellow, red rectangular petals) and extended -10 sigma70 (green) and ribosome binding sites (purple and blue) were scanned over the sequence and displayed using sequence walkers. The third sequence shows the effect of changing base 319 from an A to a C in M107L. This lowers the initiation codon from 6.3 bits to -5.5 bits, effectively removing the ribosome binding site. (PDF) [file pone.0136396.s001.pdf]

S1 Fig. Putative sigma 70 promoter and ribosome binding sites in mouse *mdr1a* cDNA. The coordinate system begins at the first base of the *mdr1a* coding region. For the first two sequences, flexible individual information models for *E. coli* sigma70 (yellow, red rectangular petals) [1] and extended -10 sigma70 (green) and ribosome binding sites (purple and blue) [2] were scanned over the sequence and displayed using sequence walkers [3]. The third sequence shows the effect of changing base 319 from an A to a C in M107L. This lowers the initiation codon from 6.3 bits to -5.5 bits, effectively removing the ribosome binding site.

1. Shultzaberger RK, Chen Z, Lewis KA, & Schneider TD (2007) Anatomy of Escherichia coli sigma70 promoters. Nucleic Acids Res 35: 771-788.
2. Shultzaberger RK, Bucheimer RE, Rudd KE, & Schneider TD (2001) Anatomy of Escherichia coli ribosome binding sites. J Mol Biol 313: 215-228.
3. Schneider TD (1997) Sequence walkers: a graphical method to display how binding proteins interact with DNA or RNA sequences. Nucleic Acids Res 25: 4408-4415.

S1 Figure

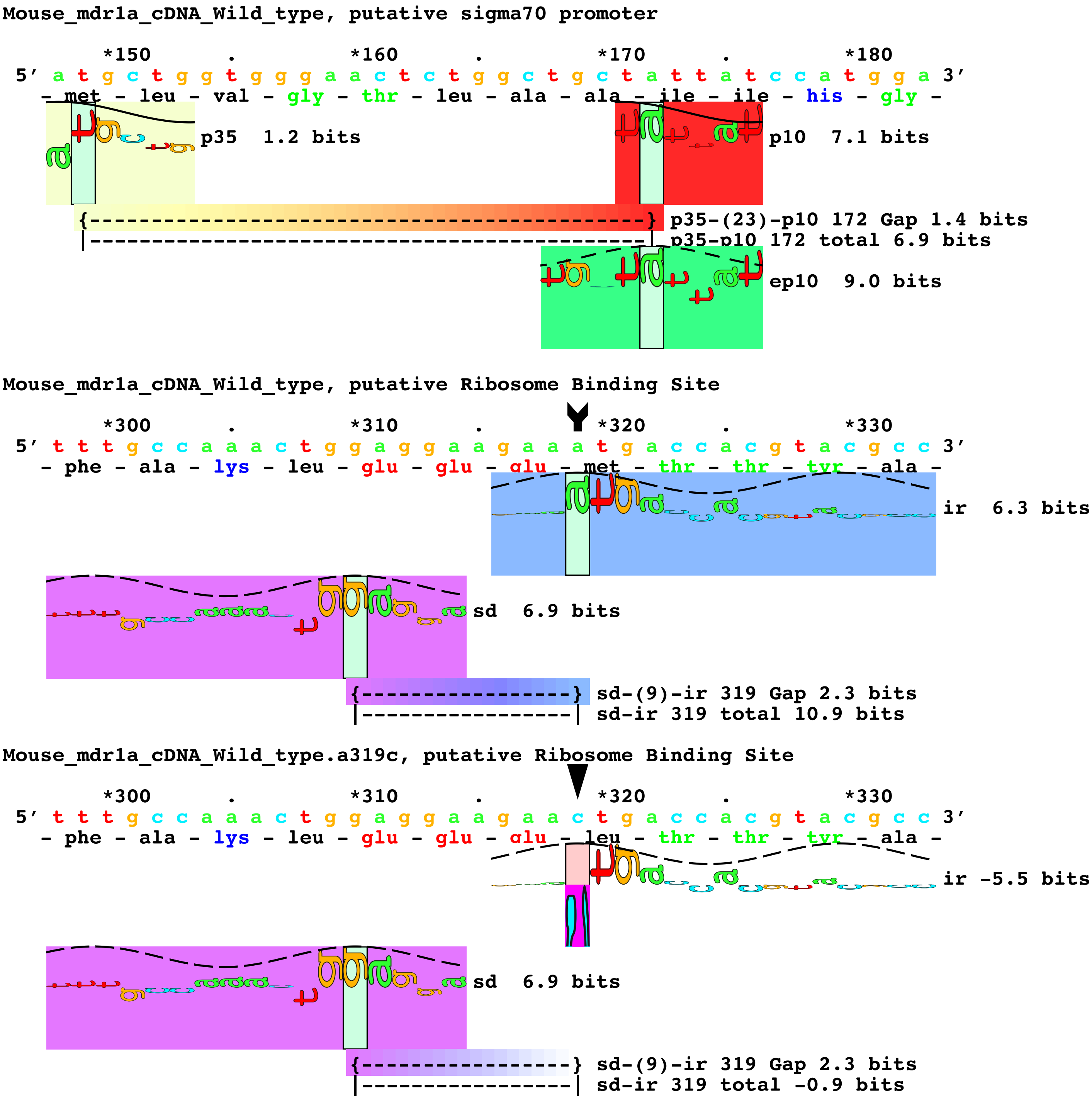
Mouse_mdr1a_cDNA_Wild_type, putative sigma70 promoter
*150 . *160 . *170 . *180
5′ a t g c t g g t g g g a a c t c t g g c t g c t a t t a t c c a t g g a 3′
- met - leu - val - gly - thr - leu - ala - ala - ile - ile - his - gly -
p35 1.2 bits
p10 7.1 bits
p35-(23)-p10 172 Gap 1.4 bits
p35-p10 172 total 6.9 bits
ep10 9.0 bits
Mouse_mdr1a_cDNA_Wild_type, putative Ribosome Binding Site
*300 . *310 . *320 . *330
5′ t t t g c c a a a c t g g a g g a a g a a a t g a c c a c g t a c g c c 3′
- phe - ala - lys - leu - glu - glu - glu - met - thr - thr - tyr - ala -
ir 6.3 bits
sd 6.9 bits
sd-(9)-ir 319 Gap 2.3 bits
sd-ir 319 total 10.9 bits
Mouse_mdr1a_cDNA_Wild_type.a319c, putative Ribosome Binding Site
*300 . *310 . *320 . *330
5′ t t t g c c a a a c t g g a g g a a g a a c t g a c c a c g t a c g c c 3′
- phe - ala - lys - leu - glu - glu - glu - leu - thr - thr - tyr - ala -
ir -5.5 bits
sd 6.9 bits
sd-(9)-ir 319 Gap 2.3 bits
sd-ir 319 total -0.9 bits
